# Supplementary material for: Prevalence and associated factors of gender-based violence for female: Evidence from school students in Nepal-A cross-sectional study
Source: PLOS Glob Public Health. 2024 Sep 26;4(9):e0003298. doi: 10.1371/journal.pgph.0003298 (PMC11426433; doi:10.1371/journal.pgph.0003298)
Supplement: S1 File — (DOCX) [file pgph.0003298.s002.docx]

# “Prevalence and Associated Factors of Gender Based Violence among High School Female Students in Brahmpuri Rural Municipality, Sarlahi”

# Manmohan Memorial Institute of Health Sciences (Affiliated TU)

**Soalteemode, Kathmandu**

**Information Sheet and Consent Form**

**Principal Investigator: Ms. Laxmi Gautam**

Namaste! I am Manisha Shah, a student pursuing bachelor’s degree of Public Health from Manmohan Memorial Institute of Health Sciences located in Soalteemode, Kathmandu. I am doing the research entitled “Prevalence and Associated Factors of Gender Based Violence among High School Female Students in Brahmapuri Rural Municipality, Sarlahi” under the supervision of principal investigator, Ms. Laxmi Gautam, Lecturer of Public Health, Manmohan Memorial Institute of Health Sciences, Kathmandu. This research study basically is trying to assess the experience of Gender Based Violence (GBV) by high school female students and the factors associated with it.

Your participation in this study is completely voluntary and will be really appreciated the research team. The information regarding the study has been provided to your parents/guardian/teacher. You can talk this over with them before you decide whether or not to participate in this study. It will take about 30 minutes or so to participate in this study, so please take your time to make your decision.

Although, you as a respondent will neither get any direct benefits nor there is any harm to participate in this study. However, the results generated from the study will be useful for planning appropriate interventions and programs to address gender based violence among adolescents. Confidentiality of your identity and information you provide will be highly maintained and shall be used solely for the study purpose.

The research proposal has been approved by the (Institutional Review Committee) IRC of Manmohan Memorial Institute of Health Sciences.

It will only be shared among the research team and nobody else. Your unbiased and genuine views and answers would be highly valued and acknowledged. If you have any queries, please feel free to contact as follows:

Name: Ms. Laxmi Gautam, Principal Investigator (Lecturer, Department of Public Health)

Phone: 9840437808

Email: [laxmi.dp26@gmail.com](mailto:laxmi.dp26@gmail.com)

Do you understand about this study and wish to participate in the study?

1. Yes, I understand about this study and willing to participate in this.
2. No, I am not willing to participate in this.

To proceed only if the participants is willing to participate in the study.

………………………………….

Student’s Signature:

Date:

**Approval Sheet from Students/Parents/Teachers**

I / my parent / teacher has read the previous page(s) of the consent form and the investigator has explained the details of the study. I/my parent /teacher understands that I am free to ask additional questions. I/my parent or guardian /teacher understands that participation in this study. The participation in this study is voluntary and I/my parent or legal guardian/ teacher may refuse to participate or may discontinue participation at any time without penalty or prejudice to the quality of care which I will receive. I/my parent or legal guardian/teacher agrees to participate in the study and have been given a copy of this form.

Student’s Signature Teacher’s Signature

**Detailed questionnaire**

| **S.N.** | **Questions** | **Answers** | **Code** | **Skip** |
| --- | --- | --- | --- | --- |
| **Section A: Demographic Information** | | | | |
| A1 | How old are you? |  |  |  |
| A2 | What is your religion? | Hindu  Muslim  Buddhist  Christian  Others (………….) | 1  2  3  4  5 |  |
| A3 | What is your ethnicity? | Brahmin/Chhetri  Janjati  Madhesi  Dalit  Others (………….) | 1  2  3  4  5 |  |
| A4 | Which type of family are you living with? | Nuclear family  Joint family  Extended family | 1  2  3 |  |
| A5 | How many members are there in your family? | …….. |  |  |
| A6 | What is place of residence? | Own house  Rent  Place of relatives | 1  2  3 |  |
| A7 | With whom you have been staying? | Parents  Husband  Relatives  Friends  Single | 1  2  3  4  5 |  |
| A8 | What is the level of education that you have been studying? | 9 class  10 class  11 class  12 class | 1  2  3  4 |  |
| A9 | What percentage did you get in last year’s final exam? | >80%  (60-80) %  (50-60) %  <50% | 1  2  3  4 |  |
| **S.N.** | **Questions** | **Answers** | **Code** | **Skip** |
| **Section A: Demographic Information** | | | | |
| A1 | How old are you? |  |  |  |
| A2 | What is your religion? | Hindu  Muslim  Buddhist  Christian  Others (………….) | 1  2  3  4  5 |  |
| A3 | What is your ethnicity? | Brahmin/Chhetri  Janjati  Madhesi  Dalit  Others (………….) | 1  2  3  4  5 |  |
| A4 | Which type of family are you living with? | Nuclear family  Joint family  Extended family | 1  2  3 |  |
| A5 | How many members are there in your family? | …….. |  |  |
| A6 | What is place of residence? | Own house  Rent  Place of relatives | 1  2  3 |  |
| A7 | With whom you have been staying? | Parents  Husband  Relatives  Friends  Single | 1  2  3  4  5 |  |
| A8 | What is the level of education that you have been studying? | 9 class  10 class  11 class  12 class | 1  2  3  4 |  |
| A9 | What percentage did you get in last year’s final exam? | >80%  (60-80) %  (50-60) %  <50% | 1  2  3  4 |  |

**Section B: Factors Related to Family**

| B1 | Are your father and mother Living together? | Yes  No | 1  2 | If yes, then go to B3 |
| --- | --- | --- | --- | --- |
| B2 | What is the reason that your father and mother are not living together? | Divorced  Mother alive father dead  Father alive mother dead  Both dead  Because of foreign employment  Others (………….) | 1  2  3  4  5  6 |  |
| B3 | What is the educational status of your father? | Illiterate (unable to read or write)  Literate (able to read or write)  Primary Education (1-8)  Secondary Education (9-12)  Higher Education (bachelors and above) | 1  2  3  4  5 |  |
| B4 | What is the educational status of your mother? | Illiterate (unable to read or write)  Literate (able to read or write)  Primary Education (1-8)  Secondary Education (9-12)  Higher Education (bachelors and above) | 1  2  3  4  5 |  |
| B5 | What is your father occupation? | Farmer  Businessman  Wage labor  Foreign employment  Government employee  Others (………….) | 1  2  3  4  5  6 |  |
| B6 | What is your mother occupation? | Farmer  Housemaker  Business women  Wage labor  Foreign employment  Government employee  Others (………….) | 1  2  3  4  5  6  7 |  |
| B7 | What is the main source of your family income? | Agriculture  Business  Govt. service  Private service  Daily wages  Foreign employment  Others (………….) | 1  2  3  4  5  6  7 |  |
| B8 | What is your monthly family income? | …… |  |  |
| B9 | Who is the decision maker of your family (food and clothing/large investment)? | Father  Mother  Grand father  Grand mother  Others (…………) | 1  2  3  4  5 |  |
| B10 | How is your relation with your Family? | Very good  Good  Bad  Very bad | 1  2  3  4 |  |
| **Substance Abuse** | | | | |
| B11 | Does any of your family members drink alcohol or other addictive substance (drugs)? If yes, then who drinks? | Yes   - Father - Mother - Elder brother - Husband - Others (………….)   No | 1  a  b  c  d  e  2 | If no, then go to B13 |
| B12 | If yes, then how many times do they drink or uses? | Frequent (Everyday - once or twice a week)  1-3 times monthly/occasionally  Sometimes (1-3 times in a year) | 1  2  3 |  |
| **Mobility** | | | | |
| B13 | Do you need permission from your family members/husband to visit your friends or close relatives? | Yes  No | 1  2 |  |
| B14 | Do you need permission from your family members/husband to visit health center or hospital? | Yes  No | 1  2 |  |
| B15 | Does your brothers also need permission to visit his friends or close relatives or health center or hospital? | Yes  No | 1  2 |  |
| **Marriage History** | | | | |
| B16 | What is your marital status? | Married  Unmarried | 1  2 | If unmarried, then go to C1 |
| B17 | At what age did you get married? | Years…. |  |  |
| B18 | What type of marriage was yours? | Arrange marriage with your consent  Love marriage  Forced marriage | 1  2  3 |  |
| B19 | Did you get married before your older brother? If so, why? | Yes  ……………………..  No | 1  2 |  |
| B20 | During your marriage dowry given to your husband’s family was demanded or voluntarily? | Voluntarily  Demanded  Don’t know  No dowry | 1  2  3  4 |  |
| B21 | Have you experience any type of physical violence (beat, kick, slap, push, shoved) or emotional violence related to dowry (scolding, blackmailing, taunting)?  **PLEASE NOTE**: Any act of violence or harassment associated with the giving or receiving of dowry at any time before, during or after the marriage.” | Yes  No | 1  2 |  |

**Section C: Experience of Gender Based Violence**

| C1 | Does your family discriminate between male and female or treats both male and female indifferently? | Yes (treat indifferently)  No (don’t treat indifferently) | | 1  2 | If no, then go to C3 |
| --- | --- | --- | --- | --- | --- |
| C2 | If yes, then in which subject does they discriminate?  **(Multiple response)** | 1. In education (e.g. you were sent in govt. school and your brother in private) 2. Regarding family respect and prestige 3. Participation in family activities (household chores) 4. Participation in social activities 5. Regarding utilization of health service 6. Mobilize family sources (pocket money) 7. Regarding entertainment (dancing, singing) 8. Choice of sports you wanted to play 9. Freedom to make friends 10. Freedom to provide your own opinion in family matters 11. Others (………..) | | | |
| **Childhood Violence** | | | | | |
| C3 | Looking back on your childhood, did you ever see or hear as any of female members of family like mother/grandmother/sister/sister in-law’s being hit, slapped, punched, shoved, kicked, or otherwise physically hurt? | Yes  No  Don’t know | | 1  2  3 |  |
| C4 | As a child, were any of your female siblings ever punched, shoved, kicked, or otherwise physically hurt differently than male siblings by your parents or guardians? | Yes  No  Don’t know  NA (no siblings) | | 1  2  3  4 |  |
| C5 | As a child or adolescent, were you ever punched, shoved, kicked, or otherwise physically hurt by your parents or guardians? | Yes  No  Don’t know | | 1  2  3 |  |
| C6 | When you were a child, did any person who was older than you touch you in a sexual way, make you touch them in sexual way, or attempt a sexual act with you? If yes then from whom you have experienced? | Yes  Family members –   - Father - Husband - Brother - Uncle - Others (……….)   Non-family members   - Teachers - Relatives - Neighbours - Strangers - Others (………...)   No | | 1  A)  a  b  c  d  e  B)  a  b  c  d  e  2 |  |
| **PHYSICAL VIOLENCE** | | | | | |
| **PLEASE NOTE:** Physical violence refers to the use of physical force against another person or group that results in physical harm, sexual or psychological harm against women or girls. It includes slapping, punching, kicking/dragging, beating/hitting with any object, cutting/ biting, shaking, shoving, pushing, throwing, and burning/chocking etc.. | | | | | |
| D1 | Have you ever experienced Physical violence differently than men in your Lifetime? | | Yes  No | 1  2 | If no, then go to E1 |
| D2 | If yes, then what type of physical violence have you faced?  **(Multiple response)** | | 1. Ever been slapped/kicked/chocked you when you talk to male friend 2. Ever been beat or pulled your hair or physically hurt you while forcing for marriage 3. Ever been slapped/ or thrown something at you that could hurt you by any of male 4. Ever beat you or physically hurt you when you go outside without taking permission 5. Ever slapped or beat or physically hurt you to do all the household works 6. Ever been pushed you, beat you or pulled your hair or hurt you more violently compared to male student at school 7. Ever beaten with fist or with something else that could hurt you 8. Ever been kicked you, dragged you or beat you up 9. Ever been chocked or burned purposely 10. Ever been threatened to use or actually used a gun, knife or other weapon against you | | |
| D3 | Have you faced any type of physical violence within 12 months? If yes then from whom you have experienced physical violence? | | Yes  Family members –   - Father - Mother - Husband - Brother - Uncle - Others (………)   Non-family members   - Teachers - Relatives - Neighbours - Strangers - Others (………)   No | 1  A)  a  b  c  d  e  f  B)  a  b  c  d  e  2 |  |
| **SEXUAL VIOLENCE** | | | | | |
| PLEASE NOTE: Sexual Violence is defined as unwanted or non-consensual sexual act through force, threat against women or girl. It includes any kind of unwanted sexual act or activity, including rape, sexual assault, sexual abuse, inappropriate touching, sexual comments etc.. | | | | | |
| E1 | Have you ever experienced sexual violence in your Lifetime? | | Yes  No | 1  2 | If no, then go to F1 |
| E2 | If yes, then what type of sexual violence have you faced?  **(Multiple response)** | | 1. Making comments about your clothing, body, behaviour, or relationships 2. Making sexual jokes or comments 3. Repeatedly asking you out on date after you have said no 4. Asking you to engage in sexual acts, such as kissing, touching, watching a sexual act, or having sex 5. Requesting for sexual photos or videos of you 6. Threatening you for saying no to a sexual request 7. Spreading rumours about your sexual life 8. Whistling or catcalling 9. Being uncomfortably close to you 10. Blocking you from moving or walking away 11. Inappropriate touching (breast, buttocks, private parts) 12. Forceful sexual contact without your consent (rape) 13. Forced into unprotected sex | | |
| E3 | Have you faced any type of sexual violence within 12 months? If yes then from whom you have experienced sexual violence? | | Yes  Family members –   - Father - Mother - Husband - Brother - Uncle - Others (………)   Non-family members   - Teachers - Relatives - Neighbours - Strangers - Others (………)   No | 1  A)  a  b  c  d  e  f  B)  a  b  c  d  e  2 |  |
| **EMOTIONAL VIOLENCE** | | | | | |
| PLEASE NOTE: Psychological Violence is the systemic destruction of women’s or girl’s self-esteem and/or sense of safety. It includes abusive teasing, threats, blackmails, controls you, taunt, scold etc.. | | | | | |
| F1 | Have you ever experienced Emotional violence in your Lifetime? | | Yes  No | 1  2 | If no, then go to G1 |
| F2 | If yes, then what type of emotional/verbal abuse you have faced?  **(Multiple response)** | | 1. Wants to know what you’re doing all the time and wants you to be in constant contact 2. Prevents or discourages you from seeing friends or family 3. Tries to stop you from going to school 4. Gets angry in a way that is frightening to you while talking with any male friend 5. Decides things for you that you should decide (like what to wear or eat) 6. Ever been scold or taunt you while forcing for marriage 7. Acts very jealous, including constantly accusing you of cheating 8. Stops you from seeing a doctor 9. Humiliates you in front of others 10. Calls you insulting names (such as “stupid,” “disgusting,” “worthless,” “whore,” or “fat”) 11. Threatens to hurt you, people you care about 12. Threatens to harm himself or herself when upset with you 13. Says things like, “If I can’t have you, then no one can” 14. Ever faced abusive teasing while walking on road or at school | | |
| F3 | Have you faced any type of emotional violence within 12 months? If yes then from whom you have experienced emotional violence? | | Yes  Family members –   - Father - Mother - Husband - Brother - Uncle - Others (………)   Non-family members   - Teachers - Relatives - Neighbours - Strangers - Others (………)   No | 1  A)  a  b  c  d  e  f  B)  a  b  c  d  e  2 |  |
| **Section G-Action taken after violence** | | | | | |
| G1 | After experiencing violence, did you report it? If yes, then whom did you report it to? | | Yes  Family members –   - Father - Mother - Husband - Brother - Sister - Grand parents - Others (………)   Other than family members   - Teachers - Police - Friends - Neighbours - Others (………)   No | 1  A)  a  b  c  d  e  f  g  B)  a  b  c  e  f  2 |  |
| G2 | If no, then what may be the reason that you did not report? | | 1. Embarrassment 2. Family prestige 3. Fear of rejection by family and friends 4. Accept violence as normal 5. Expect women or girls should only report if they have serious physical injury 6. Expect that it will damage reputation 7. Scared to report 8. No idea that it is crime   Others (………….) | | |
