# Supplementary figures and images for: Prevalence and associated factors of gender-based violence for female: Evidence from school students in Nepal-A cross-sectional study
Source: PLOS Glob Public Health. 2024 Sep 26;4(9):e0003298. doi: 10.1371/journal.pgph.0003298 (PMC11426433; doi:10.1371/journal.pgph.0003298)

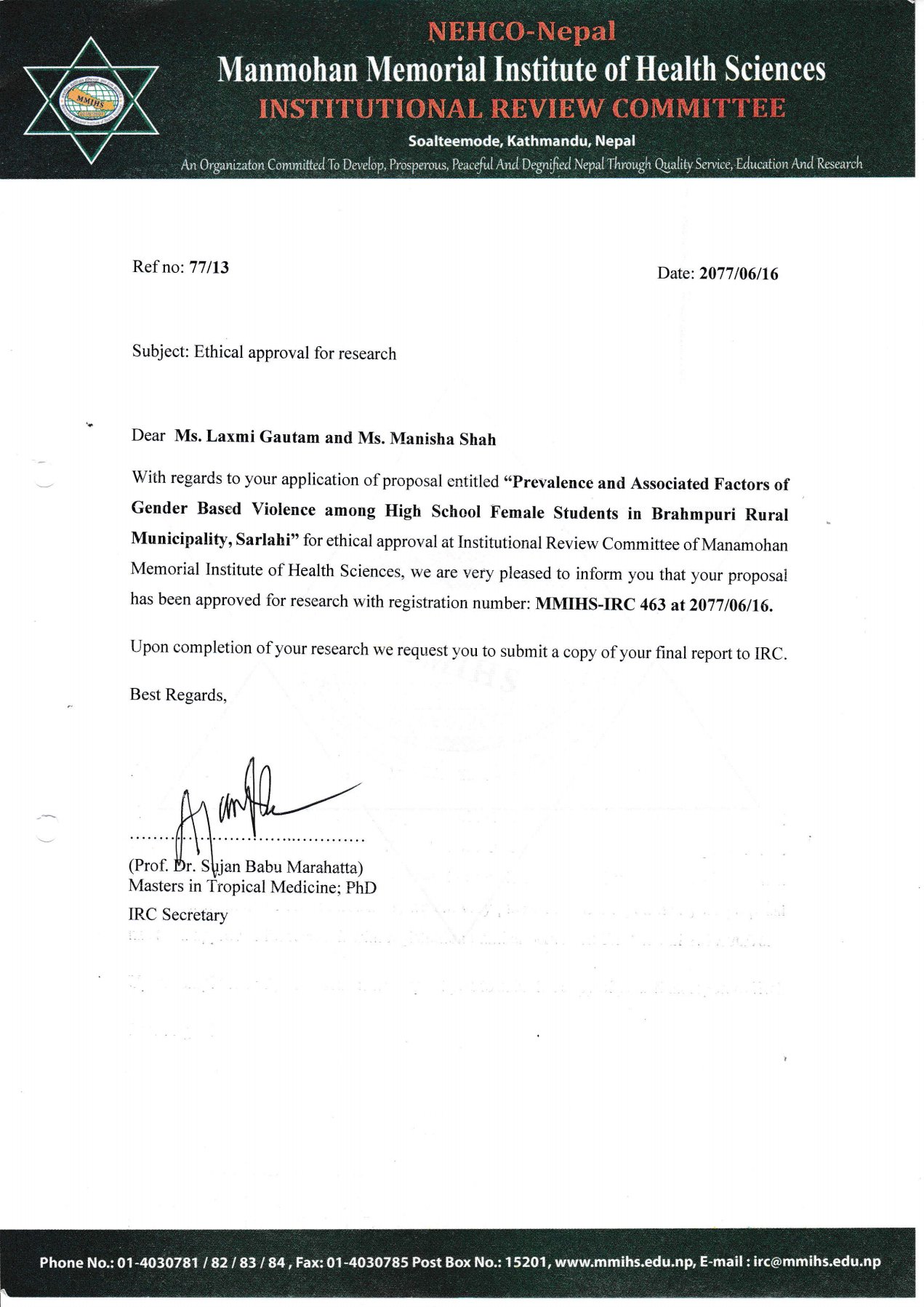

Supplement: S3 File — (DOCX) [file pgph.0003298.s004.docx]
