# Supplementary material for: Prevalence and associated factors of gender-based violence for female: Evidence from school students in Nepal-A cross-sectional study
Source: PLOS Glob Public Health. 2024 Sep 26;4(9):e0003298. doi: 10.1371/journal.pgph.0003298 (PMC11426433; doi:10.1371/journal.pgph.0003298)
Supplement: Attachment — Submitted filename: Response to Reviewers_final.docx [file pgph.0003298.s005.docx]

**Response to Reviewers:**

PGPH-D-24-01024: Prevalence and associated factors of gender-based violence for female: Evidence from school students in Nepal- a cross sectional study

Dear Editor and Reviewers,

PLOS Global Public Health

I would like to extend my sincere gratitude to the editorial and review team for the constructive suggestions and comments in our manuscript. This has undoubtedly helped us to shape our manuscript in the standards that meets the global scientific arena. Your valuable suggestions and comments were very meaningful to us. We have gone through all of the comments and have tried our best to rectify it. Our responses are as follows;

**Response to general comments:**

Questionnaire is uploaded as supplementary file.

Comment (C):

Response (R):

C-1: No action needed

C-2: No action needed

C-3: Data file uploaded as supplementary file

C-4: Grammatical and typographical errors have been corrected

**Response to the comments of Reviewer 1:**

C-1: The manuscript needs to undergo a grammatical check to correct the several grammatical errors present throughout the document.

R-1: Grammatical errors and other mistakes have been identified and corrected throughout the document as applicable. A colleague who is an expert in the English language was consulted for doing so.

C-2: The results section needs to be re-done with the tables being made more presentable in terms of alignment of results with the variables as well as proper font size to ensure the content is easily readable. The text under each table needs to describe the results in the order that the they are presented in the table.

R-2: Result section has been reviewed, reordered, and revised accordingly. So, is the table and font size.

C-3: Throughout the document, the authors should ensure that each statement is complete and makes sense. Several statements are left hanging with no clear meaning.

R-3: The whole manuscript is revised to correct such mistakes and the required revisions are made accordingly.

C-4: With regards to data availability, it is not clearly indicated where to access the data behind the finding presented in the manuscript.

R-4: The data file is uploaded as supplementary file.

**Response to the comments of Reviewer 2:**

C-1: In the Abstract session, there is need to rephrase the first sentence of the conclusion.

R-1: The first sentence of the conclusion is rephrased as;

(Different forms of GBV such as physical, sexual, and emotional acts of violence among female students were highly prevalent. However, their knowledge and awareness of confronting this issue were limited. )

C-2: Under introduction from 53 to 56 “GBV or violence against women and girls (VAWG), is a global pandemic that affects 1 in 3 women in their lifetime and 35% of women worldwide have experienced either physical and/or sexual intimate partner violence or non-partner sexual violence”. Kindly add references

R-2: Reference is added (reference no 3:: Gender Based Violence: World Bank; 2019)

C-3:3. Kindly delete (REF) in line 59

R-3: Ref is deleted.

C-4: In line 60 “Studies in India(3) , Pakistan(4) and Maldives(5) revealed that 79.6%, 38.40% and 34.6% respectively” statement is not complete

R- 4. Statement is rephrased as:

Within the South-Asian region, the magnitude of GBV varied significantly. The highest proportion (79.6%) was reported from India^(5)^followed by Pakistan (38.40)^(6)^and Maldives (34.6%).^(^**^7)^**

C-5: It would be necessary to include the definition of gender based violence in the introduction

R-5. The definition of gender based violence in the introduction is added in the beginning of the introduction as;

(The United Nations has defined GBV as, "any act of gender-based violence that results in, or is likely to result in, physical, sexual, or mental harm or suffering to women, including threats of such acts, coercion or arbitrary deprivation of liberty, whether occurring in public or in private life"**.^(1)^**)

C-6: I suggest the need to include in your introduction the impact of gender based violence (on health, mental, social well-being) on female/women, and on the society at large

R-6. The impact of gender based violence is added in the paragraph 3rd of the introduction as; (Violence against women and girls is a human rights violation and can have devastating health consequences including death. GBV has significant and long-lasting impacts on physical and mental health and injury, unintended pregnancy and pregnancy complications, sexually transmitted infections, HIV, depression, post-traumatic stress disorder, and even death. ^(12)^ Additionally, violence negatively affects women’s general well-being and prevents women from fully participating in society. ^(13)^ It impacts their families, their community, and the country at large.)

C-7: I recommend adding a stronger justification for conducting the study, for example what studies have been conducted in Nepal on GBV? What is known? What gap are you filling and why?

R-7. Justification for conducting the study is added in the last paragraph of the introduction as;

(Furthermore, patriarchal attitudes and deep-rooted stereotypes that discriminate against women remain entrenched in social, cultural, religious, socio-economic and political institutions. Despite the growing concern about violence against women, there is limited understanding regarding the underlying risk factors of GBV related to middle and late adolescence in low and middle-income countries like Nepal. This lack of evidence hinders the design and implementation of the responsive control intervention to fight against the problem. This concern was the basis for the conception of this study. Therefore, this study aimed to assess the prevalence and underlying factors associated with gender-based violence among female secondary-level students.)

C-8: Under sampling design and population, more details are needed on the sampling technique “Two schools were selected using simple random sampling and probability proportionate allocation (PPA) was used to select the study population”. Was there any exclusion criteria? Kindly include Under sampling design and population, more details are needed on the sampling technique “Two schools were selected using simple random sampling and probability proportionate allocation (PPA) was used to select the study population”. Was there any exclusion criteria? Kindly include

R-8. Sampling technique is described in detail and exclusion is also included. However, specifically, we did not have any exclusion criteria. We excluded the students who were absent during our data collection because we did not have any mechanisms to further visit for data collection of missed respondents.

The following paragraph has been revised and included in the manuscript:

(The Sarlahi district was selected purposively because percentage of women who were able to make specific decisions was lower in Madhesh Province and was lowest among all 7 provinces of Nepal (38.3%)^(10)^ Sarlahi district is one of the core areas of Madhesh Province. First of all, the list of schools was obtained from the educational section of the Brahmapuri Rural municipality. Of the total schools in that rural municipality, only four schools met our criteria (teaching grade 9^th^ to 12^th^). Out of four schools, two schools were selected using Simple Random Sampling method. Then, the list of female students from grade 9^th^ to 12^th^ was obtained from each school. Probability Proportionate Allocation (PPA) was used to select sample (female students) from selected schools, and were selected randomly using a simple random sampling method. Those students who were absent during the data collection period were excluded from the study.

C-9: Can you add references to the sources of the question from these organizations “To measure these experiences different questions were developed based on the reference provided by WHO, UNICEF and UN women and were modified to make applicable in local context of the respondents”?

R-9. References are added as per the suggestion (Ref no 16,17,18,19)

C-10: Did you explore getting informed consent from the parents of the minors? “Written informed consent was consent was taken from participants after explaining purpose of the study prior to the data collection. In case of participants below the age of 18 years, permission was taken from school principle, respective class teachers and then assent was taken from them”

R-10. We tried to get the consent from the parents of minor during pretesting, however, because of the limited resources it was not feasible. Therefore, in our final study, we did not include to take consent from their parents, however, we took permission from respective principals and class teachers, and assent was taken from the participants.

We have revised the sentence as; …………Further, permission was obtained from municipal office and each school before collecting information. Prior to the data collection, the purpose of the study was explained to the participants and written informed consent was obtained from them. In case of participants below 18 years of age, it was not feasible to reach the parents of each respondents as the resources was constrained (which was observed during pre-testing of tools). Therefore, permission was obtained from the respective school principals and class teachers, and assent was obtained from the participants. Additionally, participants were informed about the privacy and confidentiality of information, as well as the rights to withdraw and refuse from the study at any time. All other ethical guidelines were followed throughout the process of this study.

C-11: The statistical analysis was prepared appropriately. However, my concerns were on the arrangement of the result. I suggest that descriptive statistics and tables come first before inferential statistics (bivariate and multivariate analysis). For the educational status of parents I suggest having a more standardized label such as Higher/Tertiary Education, Secondary Education, Primary Education and No formal education. Was there no mothers’ with higher education from the study? Why were there no values for fathers’ education following the multivariate analysis?

R-11. The results have been rearranged as per the suggestions. Educational status of parents has been corrected. None of the mothers of the participants had higher level of education.

Also, none of the students reported having experienced GBV in the last 12 months whose father had university level of education so that this couldn't be computed in multivariate analysis. This condition as been mentioned in the footnote of table no. 4 and 5.

*(mentioned in footnote of table 4 as: ++: Cannot be computed because of zero value in one of the cells (None of the students reported having experienced GBV in the last 12 months whose father had university level of education*)

*(mentioned in footnote of table 5 as: ++: Fathers education cannot be computed because of zero value in one of the cells (None of the students reported having experienced GBV in the last 12 months whose father had university level of education****). Therefore, fathers education is not shown in the table.)***

C-12: . “To our knowledge, this is the first study to explore the prevalence and factor of GBV among the secondary school female students in Nepal”. Really?

R-12. Many studies has been done to explore the association between GBV and reproductive age women. However, we did not specifically find the study done among higher school level female students. Therefore, we assumed this study to be the nobel study in this area using aforementioned participants.

Moreover, as per the comments and suggestions from the reviewer, we have revised our discussion part and have explained the gap in the first paragraph of the discussion as;

(GBV is widely recognized as a major public health problem and is a human rights violation. It not only affects the health and dignity of the victims but also their families and generations to come. Many studies have been conducted among women of reproductive age to estimate the prevalence and establish evidence for effective interventions that address GBV.^(2, 20, 21)^ . GBV against women and girls is a product of interactions between familial and social relationships within an environment. Although there are numerous similar studies conducted among women of reproductive age, very few studies are conducted focusing particularly among adolescents girls. Therefore, to address this gap, this study was conducted which aims to explore the prevalence and factors of GBV among secondary school female students in Nepal.)

C-13: This study identified that the risk of GBV including physical, mental and sexual violence increasing among the secondary school girls where the joint family status, poor education status of parents, single parents and existence of alcohol or drug abusers in the family.” Kindly rephrase

R-13. Sentence have been rephrased.: This study revealed that the risk of GBV like physical, mental, and sexual violence among secondary-level female students is still high. Contributing factors like joint family status, poor education status of parents, single parents, and the existence of alcohol or drug abusers in the family had a significant association with the violence faced by the study population.

C-14: In discussing the prevalent of GBV is good to add implications of this findings

R-14. Implications of the findings is added in the second paragraph of discussion as;

These variations might be due to the difference in the status of girls and the gender gap. It warrants not only developing policies but also implementing these policies effectively. Women and girls friendly environments in households as well as educational institutions should be established to make the young generation free from GBV. Also, interventions focused on educating the academic and research community, parents, and students about this important issue can foster respect and equality for girls and women, and create a safe environment.

C-15: However, there is a need for copy editing to correct few typographical or grammatical at revision.

R-15: The typographical and grammatical errors are corrected.

We attempted to rectify all comments and suggestions that had been raised by the reviewers. We hope that all the concerns have been addressed. We are very grateful for your time and feedback.

Thank you very much.

Sincerely

Laxmi Gautam
